# Short-Term Consequences of Angiographically-Confirmed Coronary Stent Thrombosis

**DOI:** 10.1371/journal.pone.0077330

**Published:** 2013-10-15

**Authors:** Christine G. Kohn, Jeffrey Kluger, Meena Azeem, Craig I. Coleman

**Affiliations:** 1 Department of Pharmacy Practice, University of Connecticut School of Pharmacy, Storrs, Connecticut, United States of America; 2 The Evidence-Based Practice Center, Hartford Hospital, Hartford, Connecticut, United States of America; 3 Department of Cardiology, Hartford Hospital, Hartford, Connecticut, United States of America; S.G.Battista Hospital, Italy

## Abstract

**Objectives:**

To conduct a meta-analysis to quantify the real-world incidence of in-hospital or 30-day death or myocardial infarction (MI), and angiographically-confirmed ST-related treatment costs.

**Background:**

The short-term clinical and economic consequences of coronary stent thrombosis (ST) are thought to be significant.

**Methods:**

We searched MEDLINE, Embase and Scopus from January 2000-July 2012 to identify observational/registry studies that evaluated a cohort of ≥25 patients experiencing angiographically-confirmed thrombosis of a drug-eluting or bare-metal stent, required the use of dual-antiplatelet therapy for guideline-recommended durations, and reported incidences of in-hospital or 30-day death or MI and/or ST-related treatment costs. Incidences and costs from each study were pooled using random-effects meta-analysis.

**Results:**

Twenty-three studies were included. Of the 13 studies reporting in-hospital outcomes, 12 (N=8,832 STs) reported mortality data, with the pooled incidence rate estimated to be 7.9%, 95%CI=5.4%-11.3%, I^2^=86%. Ten studies (N=1,294 STs) reported 30-day death, with a pooled incidence of 11.6%, 95%CI=8.8%-15.1%, I^2^=55%. Patients experiencing early ST (within 30-days of implant) had higher in-hospital and 30-day mortality than those experiencing very-late ST (interaction p<0.04 for both). Stent type had no significant effect on in-hospital or 30-day mortality. In the 5 studies (N=542 STs) and 3 studies (N=180 STs) reporting in-hospital and 30-day MI, respectively, the pooled incidence rates were 6.1%, 95%CI=2.1%-16.2%, I^2^=88% and 9.5%, 95%CI=3.8%-22.0%, I^2^=65%. One study reported costs associated with ST, estimating the median/patient cost of hospitalization to treat early ST at $11,134 (in 2000US$).

**Conclusions:**

Regardless of stent type used, the short-term consequences of coronary ST appear significant.

## Introduction

The clinical consequences for patients experiencing coronary stent thrombosis (ST) are thought to be dire [1-3]. A pooled analysis of six multicenter bare-metal coronary stent clinical trials demonstrated patients experiencing an angiographically-confirmed ST had a 30-day incidence of the combined endpoint of death or myocardial infarction (MI) of 64% [3]. However, the incidence of death and MI observed in this analysis may not accurately reflect what would be seen in a real-world population. In addition to the clinical consequences, the occurrence of ST has been associated with a significant economic impact as well, as depicted by retrospective study estimating the median total hospital cost to treat a ST to be upwards of $11,134 per patient (in 2000 U.S. dollars) [4]. 

As no such systematic review and meta-analysis of real-world data/studies has been published to date, we sought to conduct a meta-analysis to better quantify the real-world incidence of in-hospital or 30-day death or MI and/or ST-related treatment costs derived from observational studies and coronary stent registries.

## Methods

We followed the Preferred Reporting Items for Systematic Reviews and Meta-Analyses (PRISMA) guidelines for the reporting of this systematic review and meta-analysis [5].

A systematic literature search using the Medline (January 2000-July 2012), Embase (January 2000-July 2012) and Scopus (January 2000-July 2012) computerized databases was conducted. The search began at the year 2000 (inclusive) to limit the identification of studies using outdated practices (outdated stents and implantation practices, or no/suboptimal use of guideline recommended dual antiplatelet therapy (DAPT)). The complete search strategy for Medline is available in Appendix S1 in File **S1**. Review of the reference sections of eligible studies as well as review articles was also performed to identify additional relevant studies. 

To be included in this systematic review and meta-analysis, studies had to 1) be an observational or registry study (to assess “real-world” outcomes), 2) evaluate a cohort of ≥25 patients experiencing angiographically-confirmed (definite) thrombosis of a drug-eluting stent (DES) or bare-metal stent (BMS) [6], 3) require the use of DAPT according to guideline recommendations of the time, 4) provide data on the incidence of in-hospital or 30-day death or MI (new or re-infarction, not counting the ST-defining ischemia) and/or ST-related treatment costs, and 5) be a full-text publication in the English language. In studies with more than one published report on the same study population, the most recent publication was selected for analysis to avoid double-counting participants, although previous publications were reviewed to supplement for missing/additional data where applicable. When studies with non-identical but overlapping populations were identified, the most inclusive publication was selected for analysis. When needed, authors of identified studies were contacted for clarification or additional data. In all situations, two investigators (CGK and MA) determined study eligibility independently, with disagreements resolved by discussion or by a third investigator (CIC).

Two investigators (CGK and CIC) performed all data extraction. Data collected for each study included author and publication year, number of STs identified, timing of data collection (prospective or retrospective), inclusion/exclusion criteria, stent type(s) evaluated (BMS and/or DES), country where study was conducted, time frame for patient/case inclusion, definition of ST [6], timing of ST (acute, sub-acute, early, late, very late) [6], use of DAPT at time of ST, funding source and role, and the incidence of in-hospital or 30-day death or MI and ST-related treatment costs. 

The validity of included studies for assessing the incidence of in-hospital or 30-day death or MI and thrombosis-related treatment costs was assessed using the assessment tool included in Appendix S2 in File **S1**. The tool assessed the following attributes : (1) whether post-ST outcomes and costs were the primary endpoints of the identified study, 2) whether the inclusion/exclusion criteria for the study were clearly described, 3) adequacy of sampling by assessing whether consecutive and unselected patients/ST cases were evaluated, 4) use of prospective data collection, 5) whether the methods of data collection for ST and outcomes data, completeness of follow-up and reasons for loss-to-follow-up were clearly described, 6) the use of a standard/acceptable definition of ST (Appendix S3 in File **S1**) and MI [6], 7) independent adjudication of ST and MI diagnoses, 8) whether studies’ discussion and conclusion sections were consistent with their results, 9) discussion of previous studies evaluating post-ST outcomes and study limitations were provided, and 10) study funding and the role of the funder in the research was described. We did not attempt to give a summary validity rating to each individual study in this systematic review. Study validity was conducted for informational purposes only, and was not used as an exclusion criterion or to perform any type of statistical adjustment. 

Numerators (n = number of patients reporting the outcome of in-hospital or 30-day mortality or MI) and denominators (N = total number of STs evaluated) were extracted from each study in order to compute incidences with accompanying 95% confidence intervals (CIs). Proportions were then pooled using DerSimonian-Laird weights (a random-effects model). Between-study heterogeneity was assessed using the I^2^ statistic with a threshold of 50% used to define an important degree of heterogeneity. To assess for the potential for publication bias, we reviewed Egger’s weighted regression statistic *p* values (with *p* <0.05 suggesting a higher likelihood of publication bias). As only one study reporting ST-related treatment costs was identified, no meta-analysis was performed for this endpoint.

 We conducted various subgroup analyses to examine the effect of ST timing (early, late and very late) and stent type (BMS and DES only analyses) on the meta-analysis’ results. We considered *P* <0.05 statistically significant for all analyses. All analysis was conducted using Comprehensive Meta-Analysis version 2 (Biostat, Englewood, NJ).

## Results

**Figure 1:**
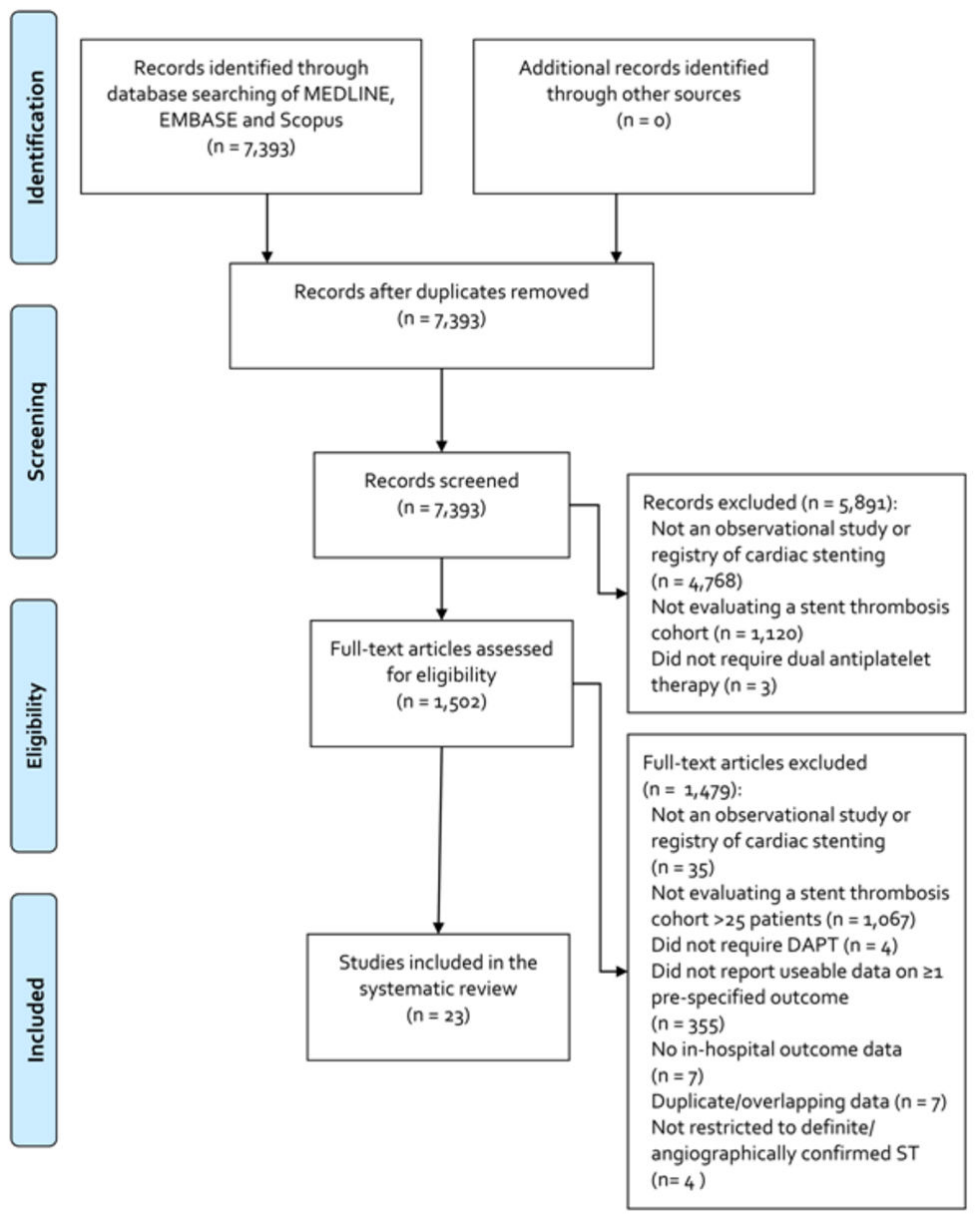
PRISMA for In-Hospital and 30-Day Post-Stent Thrombosis Mortality and Myocardial Infarction and Treatment Cost Meta-Analyses.

The initial search yielded a total of 7,393 non-duplicate citations. Reasons for exclusion during title and abstract and full-text article review are described in Figure **1**. A total of 23 studies were included in this systematic review and meta-analysis [4,7-28], of which 13 were included in the in-hospital analyses and 10 in the 30-day analyses (Tables **1** and **2**). More specifically, 12 studies had data on in-hospital death [4,7,10,12,14-16,21,24,25,27,28] and 5 studies reported data on in-hospital MI [10,12,16,22,27], 10 studies reported on 30-day death [8,9,11,13,17-20,23,26] , 3 on 30-day MI [8,9,11] and one evaluated the cost of the index hospitalization due to angiographically-confirmed ST [4]. 

**Table 1:** Characteristics of Included Studies for the In-Hospital Post-Stent Thrombosis Mortality and Myocardial Infarction Meta-Analyses.

**Author, Year (N STs)**	**Data Recording**	**Inclusion Criteria**	**Stent Type**	**Country of Conduct**	**Inclusion Years**	**Definition of ST**	**Timing of ST**	**APT at ST Diagnosis**	**Funding**
Armstrong 2012 (N=7,315)	R	Patients in The CathPCI Registry presenting with a ST and ACS (64% presented with STEMI)	DES=56%; BMS=23%; Unknown=21%	>100 centers in the United States	2009-2010	Definite	EST=20%; LST=19%; VLST=61%	NR	NR
Chechi 2008 (N=92)	R	Patients presenting with STEMI deemed a result of ST	BMS=24%; DES=76%	1 center in Italy	2004-2007	Definite	EST=64%; LST=15%; VLST=21%	DAPT=67%	NR
Daemen 2007 (N=152)	Unclear	Patients undergoing SES or PES implantation and developing ST (45% presenting with MI)	DES=100%	2 centers in the Netherlands and Switzerland	2002-2005	Definite	EST=60%; LST/VLST=40%	DAPT=61%	Academic
De la Torre-Hernandez 2008 (N=301)	P	Any patients presenting with ST (37% presented with STEMI)	DES=100%	20 centers in Spain	2002-2007	Definite	AST=8%; SAST=42%; LST=50%	DAPT=68.4%	NR (not Industry)
Del Pace 2010 (N=41)	R	Consecutive patients presenting with ST (39% presented with STEMI, 78% with acute MI)	BMS=29%; DES=71%	1 center in Italy	2005-2006	Angiographically or autopsy confirmed with ACS or sudden cardiac death (all cases were confirmed)	AST=12%; SAST=56%; LST/VLST=32%	DAPT=80%	NR
Ergelen 2010 (N=118)	R	Patients presenting with STEMI deemed a result of ST	NR	1 center in Turkey	2003-2008	Definite	SAST=35%; LST=31%; LST=35%	NR	None
Lee 2010 (N=30)	Unclear	Patients presenting with VLST and acute MI and undergoing IVUS (78% presented with STEMI)	BMS=23%; DES=77%	1 center in South Korea	2004-2009	Definite	VLST=100%	DAPT=7%	Foundation
Lemesle 2009 (N=91)	R	Patients previous undergoing DES implantation and presenting with ST (75% presented as STEMI, 100% ACS)	DES=100%	1 center in the United States	2003-2008	Definite	NR	NR	NR
Pinto Slottow 2008 (N=84*)	R	Patients experiencing a ST any time after PCI (38% experiencing a MI during ST admission)	DES=100%	1 center in the United States	2003-2007	Definite	EST=62%; LST=20%; VLST=18%	ASA=90%; CLO=82%	NR
Reynolds 2002 (N=26)	R	All patients presenting with ST and complete billing records	BMS=100%	1 center in the United States	1998-2000	Angiographically confirmed	SAST=100%	NR	Industry
van Werkum 2009 (N=431)	I	Consecutive patients presenting with ST	BMS=62%;DES=35%; Both:4%	3 centers in the Netherlands	2004-2007	Definite	AST=33%; SAST=41%; LST=13%; VLST=13%	ASA=87%; CLO=69%	Industry
Wenaweser 2005 (N=95)	R	All patients presenting with ST and undergoing emergency PCI	BMS=100%	1 center in Switzerland	1995-2003	Definite	AST=11%; SAST=64%; LST=25%	CLO/TIC=76%	NR
Yeo 2011 (N=165**)	P/R	Patients presenting with ST (63% presenting with STEMI, 91% with MI)	NR	Multiple centers in the United States (California)	2005-2010	Definite	AST=4%; SAST=22%; LST=18%; VLST=50%	DAPT=41%	NR

ACS=acute coronary syndrome; APT=antiplatelet therapy; ASA=aspirin; AST=acute stent thrombosis; BMS=bare metal stent; CLO=clopidogrel; DAPT=dual antiplatelet therapy; DES=drug-eluting stent; EST=early stent thrombosis; I=indeterminate; IVUS=intravascular ultrasound; LST=late stent thrombosis; MI=myocardial infarction; N=number of stent thromboses; NR=not reported; P=prospective; PCI=percutaneous coronary intervention; R=retrospective; SAST=sub-acute stent thrombosis; ST=stent thrombosis; STEMI=ST-segment elevation myocardial infarction; TIC=ticlopidine; VLST=very late stent thrombosis

*83 patients had 84 Definite STs; ** 153 patients had 165 Definite STs

**Table 2:** Validity Assessment of Included Studies for the In-Hospital Post-Stent Thrombosis Mortality and Myocardial Infarction Meta-Analyses.

**Study, Year (N STs)**	**Primary Endpoint**	**Inclusion/Exclusion**	**Consecutive & Unselected**	**Prospective**	**Methods Described**	**Follow-Up**	**Endpoint Validity**	**Adjudication**	**Discussion/ Conclusion**	**Previous Studies**	**Limitations**	**Funding Described**
Armstrong 2012 (N=7,315)	Y	Y	Y	N	Y	Y	Y	I	Y	Y	Y	N
Chechi 2008 (N=92)	Y	Y	N	N	Y	Y	Y	I	Y	N	Y	N
Daemen 2007 (N=152)	N	Y	N	I	Y	Y	N	Y	NA	NA	Y	Y
De la Torre-Hernandez 2008 (N=301)	Y	Y	I	Y	Y	Y	Y	I	N	N	Y	N
Del Pace 2010 (N=41)	N	Y	Y	N	Y	Y	Y	I	NA	NA	Y	N
Ergelen 2010 (N=118)	N	N	I	N	Y	Y	Y	I	NA	NA	Y	Y
Lee 2010 (N=30)	N	Y	N	I	N	Y	Y	I	NA	NA	Y	Y
Lemesle 2009 (N=91)	N	Y	N	N	Y	Y	Y	Y	Y	NA	Y	N
Pinto Slottow 2008 (N=84*)	Y	Y	N	N	Y	Y	Y	Y	Y	N	Y	N
Reynolds 2002 (N=26)	Y	Y	N	N	Y	N	N	I	Y	NA	Y	N
van Werkum 2009 (N=431)	Y	Y	Y	I	Y	Y	Y	Y	Y	Y	Y	Y
Wenaweser 2005 (N=95)	Y	Y	Y	N	Y	Y	Y	I	Y	Y	Y	N
Yeo 2011 (N=165**)	Y	N	I	I	Y	Y	Y	Y	Y	Y	Y	N

I=indeterminate; N=no; Y=yes; NA=not applicable

*83 patients had 84 Definite STs; ** 153 patients had 165 Definite STs

Included studies were published between 2000 and 2012 (patient inclusion between 1993 and 2010) and followed as few as 23 and as many as 7,315 ST cases. The patients experiencing angiographically-confirmed ST came from various geographic regions, 30% in the United States as well as France, Hungry, Israel, Italy, Japan, Netherlands, South Korea, Spain and Switzerland. Eleven studies included a mix of patients with both BMS and DES, 10 studies included a single stent type and two did not report stent type. While inclusion criteria required all patients to have angiographically-confirmed ST, a majority of included studies (87%) required patients meet the Academic Research Consortium’s (ARC’s) criteria for “definite” ST [acute myocardial ischemia (ECG major ST abnormality or any biomarker elevation) and angiographic or autopsy evidence of stent occlusion or thrombus] or some close variation (two of the three studies not meeting the ARC definite definition were conducted and published prior to the definitions creation) [6]. Most studies included cases of angiographically-confirmed ST regardless of their timing (early, late or very late) and thus had a mixture of such cases; however, 10 studies reported in-hospital or 30-day data for those experiencing early ST, 6 studies reported data for those experiencing late ST, and 4 for very late ST. Three studies stated they were funded by an industry partner, two and one study, respectively, were funded by an academic institution and a foundation, two studies reported being unfunded, and the remainder did not provide information of funding.

Study validity assessment as it pertains to answering this review’s aims are depicted in Tables **3** and **4**. The most common validity concerns noted for included studies were: 1) not planning to follow post-ST outcomes as a primary objective of the study (studies were designed for a different purpose), 2) failure to include consecutive and unselected populations, 3) not collecting data in a prospective fashion, 4) not independently adjudicating ST and MIs, and 5) not adequately describing funding or the role of the funder in the research. 

**Table 3:** Characteristics of Included Studies for the 30-Day Post-Stent Thrombosis Mortality and Myocardial Infarction Meta-Analyses.

**Author, Year (N STs)**	**Data Recording**	**Inclusion Criteria**	**Stent Type**	**Country of Conduct**	**Inclusion Years**	**Definition of ST**	**Timing of ST**	**APT at ST Diagnosis**	**Funding**
Becker 2009 (N=47)	P	Patients experiencing STEMI as a consequence of ST	Both DES and BMS; % NR	1 center in Hungary	2003-2005	Definite	AST= 11%; SAST/LST=89%	CLO/TIC=90%;ASA=87%	NR
Burzotta 2008 (OPTIMIST) (N=110)	P/R	Any patient with angiographically confirmed ST, resting angina or new ischemic ECG changes or cardiac biomarker elevations (73% STEMI at presentation)	BMS=50%; DES=39%; Unknown=11%	11 centers in Italy	2005-2006	Definite	EST=72%; LST=20%; VLST=8%	NR	Unfunded
Cheneau 2003 (N=27*)	P	Patients experiencing SAST within 7 days of IVUS-guided stent implantation (excluded MI within 1 month of intervention)	BMS=100%	1 center in the United States	1993-2002	Recurrent ischemia and documented stented vessel occlusion within 7 days of index PCI	SAST=100%	DAPT=100%	NR
Dannenberg 2009 (N=29)	I	Patients who underwent angioplasty and were readmitted with a diagnosis of ST (53% MI at presentation)	DES=25%; BMS=75%	1 center in Israel	2004-2006	Definite	Between 2 days and 3 years after ST; LST=10%	CLO=38%; ASA=66%	NR
Heestermans 2010 (N=201)	I	Consecutive patients stented for STEMI and presenting with ST	BMS=28%; DES=70%; Unknown=2%	3 centers in the Netherlands	2004-2007	Definite	AST=48%; SAST=52%	CLO=78%	NR
Kuchulakanti 2006 (N=38)	P	All patients treated with DES	DES=100%	1 center in the United States	2003-2004	Definite	AST=13%; SAST=66%; LST=21%	DAPT=63%	NR
Kimura 2010 (RESTART) (N=611)	R	Patients undergoing PCI with sirolimus-eluting stent implantation (69% STEMI, 23% NSTEMI/UA at presentation)	DES=100%	543 centers in Japan	2004-2008	Definite	EST=53%; LST=17%; VLST=30%	DAPT=55%	Industry
Le Feuvre 2008 (n=52)	I	Patients experiencing ST of a BMS or DES	BMS=69%; DES=31%	1 center in France	2003-2007	Definite	EST=73%; LST=13%; VLST=13%	DAPT=79%	NR
Mahmoud 2011 (N=113)	P	All patients undergoing PCI at a single institution (85% presenting with STEM)	BMS=23%; DES=77%	1 center in the Netherlands	2002-2010	Definite	EST=52%; LST=25%; VLST=23%	ASA=61%; CLO=50%	NR
Wenaweser 2008 (N=192)	I	Patients undergoing SES or PES implantation and developing ST (68% ACS at presentation)	DES=100%	2 centers in Switzerland and the Netherlands	2002-2005	Definite	EST=48%; LST=16%; VLST=69%;	DAPT=56%	Academic

ACS=acute coronary syndrome; APT=antiplatelet therapy; ASA=aspirin; AST=acute stent thrombosis; BMS=bare metal stent; CLO=clopidogrel; DAPT=dual antiplatelet therapy; DES=drug-eluting stent; ECG=electrocardiogram; EST=early stent thrombosis; I=indeterminate; IVUS=intravascular ultrasound; LST=late stent thrombosis; MI=myocardial infarction; N=number of stent thromboses; NR=not reported; NSTEMI=non-ST-segment-elevation myocardial infarction; P=prospective; PCI=percutaneous coronary intervention; R=retrospective; SAST=sub-acute stent thrombosis; ST=stent thrombosis; STEMI=ST-segment elevation myocardial infarction; TIC=ticlopidine; UA=unstable angina; VLST=very late stent thrombosis

* Only 23 ST patients analyzed

**Table 4:** Validity Assessment of Included Studies for the 30-Day Post-Stent Thrombosis Mortality and Myocardial Infarction Meta-Analyses.

**Study, Year (N STs)**	**Primary Endpoint**	**Inclusion/Exclusion**	**Consecutive & Unselected**	**Prospective**	**Methods Described**	**Follow-Up**	**Endpoint Validity**	**Adjudication**	**Discussion/ Conclusion**	**Previous Studies**	**Limitations**	**Funding Described**
Becker 2009 (N=47)	Y	Y	N	Y	Y	Y	I	I	Y	Y	N	N
Burzotta 2008 (OPTIMIST) (N=110)	Y	Y	Y	I	Y	Y	Y	Y	Y	Y	Y	Y
Cheneau 2003 (N=27*)	N	Y	N	Y	Y	N	N	Y	NA	NA	Y	N
Dannenberg 2009 (N=29)	N	Y	N	I	Y	Y	Y	I	NA	NA	N	N
Heestermans 2010 (N=201)	Y	Y	N	I	Y	Y	Y	Y	Y	Y	Y	N
Kimura 2010 (RESTART) (N=611)	Y	Y	N	N	Y	Y	Y	Y	N	N	Y	N
Kuchulakanti 2006 (N=38)	Y	Y	N	Y	Y	Y	Y	Y	Y	Y	Y	N
Le Feuvre 2008 (n=52)	Y	Y	Y	I	N	Y	Y	I	Y	Y	N	N
Mahmoud 2011( N=113)	N	Y	Y	Y	Y	Y	Y	Y	Y	NA	Y	N
Wenaweser 2008 (N=192)	N	Y	N	I	Y	Y	N	Y	Y	Y	Y	Y

I=indeterminate; N=no; Y=yes; NA=not applicable

**Figure 2:**
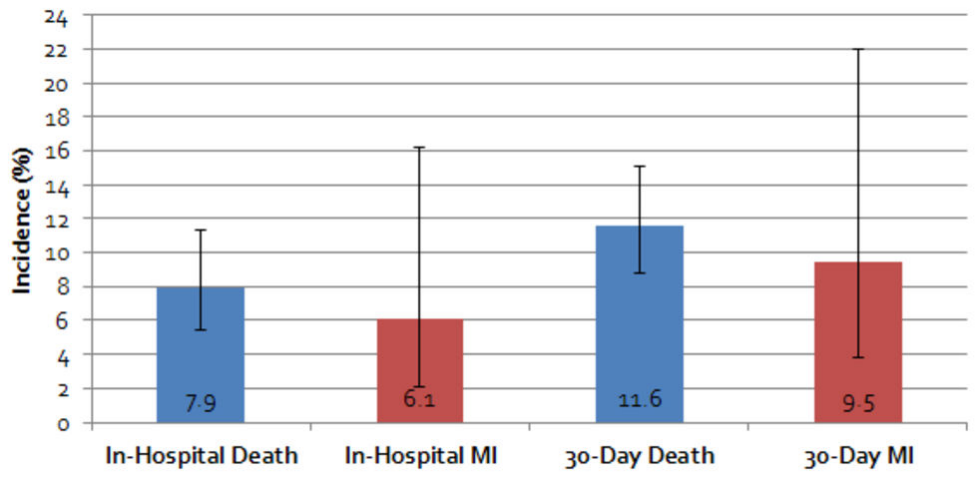
Pooled Incidence Rates and 95% Confidence Intervals for In-Hospital and 30-Day Mortality and Myocardial Infarction.

Raw incidence data for the death and MI outcomes are provided in Table **5**. Among the 12 studies (N=8,832 angiographically-confirmed STs) reporting in-hospital mortality (median incidence: 7.0%, range: 3.8%-19.5%), the pooled incidence rate was estimated to be 7.9%, 95%CI=5.4%-11.3% (Figure **2**). A significant amount of statistical heterogeneity was noted in this analysis (I^2^=86%) and a higher likelihood of publication bias was noted (Eggers p=0.04). At 30-days, the pooled incidence of mortality based upon 10 studies (N=1,294) (median incidence: 10.6%, range: 3.4%-26.9%) was 11.6%, 95%CI=8.8%-15.1%. Again, a significant amount of statistical heterogeneity (I^2^=55%) was noted; however, a lower likelihood of publication bias was observed (p=0.56). Subgroup analysis suggested patients experiencing early ST had higher in-hospital and 30-day mortality than those experiencing very late ST (interaction p<0.04 for both); while stent type (bare-metal vs. drug-eluting) had no significant effect on in-hospital or 30-day mortality rates (Table **6**)**.**

**Table 5:** Incidences of In-Hospital and 30-Day Death and Myocardial Infarction from Identified Studies.

**Study, Year**	**In-Hospital Death**	**In-Hospital Myocardial Infarction**	**30-Day Death**	**30-Day Myocardial Infarction**
Armstrong 2012	317/7315	---	---	---
Becker 2009	---	---	3/47	2/47
Burzotta 2008	---	---	13/110	8/110
Chechi 2008	15/86	7/86	---	---
Cheneau 2003	---	---	2/23	5/23
Daemen 2007	11/152	2/152	---	---
Dannenberg 2009	---	---	1/29	---
De la Torre-Hernandez 2008	35/301	---	---	---
Del Pace 2010	8/41	---	---	---
Ergelen 2010	12/118	5/118	---	---
Heestermanns 2010	---	---	19/201	---
Kimura 2010	---	---	60/489	---
Kuchulakanti 2006	---	---	6/38	---
Le Feuvre 2008	---	---	14/52	---
Lee 2010	2/30	---	---	---
Lemesle 2009	---	22/91	---	---
Mahmoud 2011	---	---	15/113	---
Pinto Slottow 2008	4/84	---	---	---
Reynolds 2002	1/26	---	---	---
van Werkum 2009	26/431	---	---	---
Wenaweser 2008	---	---	13/192	---
Wenaweser 2005	7/95	4/96	---	---
Yeo 2011	6/153	---	---	---

MI=myocardial infraction

**Table 6:** Results of Subgroup and Sensitivity Analyses of Post-Stent Thrombosis Mortality.

**Characteristic**	**In-Hospital Death (N Studies); Incidence (95%CI)**	**Interaction *P* Value**	**30-Day Death (N Studies**)**; Incidence (95%CI**)	**Interaction *P* Value**
ST Timing				
EST only	N=5; 10.7% (7.1-16.0)*	Referent	N=5; 12.3% (8.7-17.1)	Referent
LST only	N=4; 6.4% (3.4-11.8)*	0.18	N=2; 13.3% (7.1-23.8)	0.83
VLST only	N=2; 3.6% (3.1-4.2)	<0.001	N=2; 26.2% (3.5-10.9)	0.04
Stent Type				
BMS only	N=2; 6.8% (3.4-13.0)	Referent	N=3; 18.2% (10.9-28.7)	Referent
DES only	N=3; 8.4% (5.1-13.5)*	0.62	N=5; 12.2% (7.9-18.3)*	0.22

* I^2^>50%

For the 5 studies (N=542 angiographically-confirmed STs) and 3 studies (N=180 angiographically-confirmed STs) reporting in-hospital (median incidence: 4.2%, range: 1.3%-24.2%) and 30-day MI (median incidence: 7.3%, range: 4.3%-21.7%), respectively, the pooled incidence rates were 6.1%, 95%CI=2.1%-16.2% (I^2^=88%) and 9.5%, 95%CI=3.8%-22.0%, (I^2^=65%). A higher likelihood of publication bias was suggested by the Eggers weight regression statistic p-value for the in-hospital MI endpoint (p=0.002), but not the 30-day endpoint (p=0.97). 

Only one study reported costs associated with angiographically-confirmed ST, estimating the median/mean± standard deviation per patient cost of hospitalization to treat early ST at $11,134/$17,400±17,758 (in 2000US$). The majority of these costs were associated with the cardiac catheterization laboratory ($5,496±2,833) followed by time spent in the intensive care unit (mean length-of-stay=1.8±2.3 days, associated costs= $3,692 ± 5,229). 

## Discussion

To our knowledge, this is the first meta-analysis with the aim of better quantifying the real-world incidence of in-hospital or 30-day death or MI, as well as, angiographically-confirmed ST-related treatment costs to be conducted. Our meta-analyses suggested angiographically-confirmed ST is associated with significant consequences; with nearly 8 out of every 100 patients experiencing an ST dying and 6 of 100 suffering a new or recurrent MI (not counting the ST-defining event) in-hospital. The rate of these same major adverse cardiovascular events increased to greater than 11% and 9%, respectively by 30-days. While we identified only a single study [4] describing costs associated with treating angiographically-confirmed ST, available data suggested treating ST may be associated with a significant economic burden to the healthcare system. 

There are multiple definitions of ST that are utilized in studies evaluating the incidence or outcomes of ST [6]. The “definite” definition requires angiographic or autopsy-confirmation, and therefore maximizes specificity (minimizes false positive diagnoses), but at the cost of underestimating the incidence (or missing cases) of ST. The supplementary use of more clinical definitions of ST (i.e., “probable” and “possible”) can add sensitivity (misses fewer cases of ST); however, their utility is highly dependent on the quality of data available to adjudicate ST events [6]. For a number of reasons, our meta-analysis only included studies reporting outcomes associated with “definite” or angiographically-confirmed ST. Since the large majority of studies did not report clinical ST along with definite/ angiographically-confirmed ST, we wanted to avoid pooling studies using disparate definitions to minimize statistical heterogeneity in our analyses. Moreover, since our meta-analysis was focusing on real-world observational studies, many of which were retrospective and failed to independently adjudicate outcomes, we had concerns about a higher risk of misclassification bias in these studies. It must be stressed, however, our restriction to angiographically-confirmed ST (to improve internal validity) means that patients not surviving to angiography were not commonly evaluated in included studies, and our analysis likely underestimates the true negative consequences of ST. 

To our knowledge, the only attempt to systematically assess outcomes following ST is the 2001 paper pooling 6 multi-centered BMS randomized trials by Cutlip and colleagues [3]. That meta-analysis followed a total of 45 patients diagnosed with angiographic ST (0.7% of the pooled population), and reported a lower incidence of 30-day death compared to our meta-analysis (6.7% vs. 11.6%). One explanation for this discrepancy between Cutlip’s and our meta-analysis may be their use of randomized trial patient data which is likely not generalizable to the more expansive and sicker populations commonly treated with stents in real-world settings. Cutlip’s use of only BMS trials cannot be ruled out as an explanation for differences in 30-day morality estimates, since recent analyses suggest DES use in the modern era may result in better outcomes then BMS use [29,30]. In contrast to the 30-day death outcome, our meta-analysis found a lower incidence of 30-day MI (9.5%) then the aforementioned analysis (15.6% Q-wave, 44.4% non-Q-wave). This finding may be a result of detection bias (patients were more aptly followed for MI in the randomized trial setting than in observational studies) or it may be a result of differences in what timing of MI was counted. More specifically, ST studies appear to vary in how they include post-ST MI, with some studies including the initial ST-defining ischemic event and some excluding it; counting only new or reinfarction in their in-hospital and 30-day MI outcomes. Because most observational studies of ST include only patients who present with acute coronary syndrome, counting the ST-defining ischemic event results in much higher estimates of MI incidence, often as high as 85%-95% by 30-days [31]. Of note, while most of the studies we identified in our systematic review exclude the ST-defining ischemic event from their MI endpoint, not all did. Thus, we had to exclude a small number of studies [23,31] from our MI meta-analysis because they either stated they included the ST-defining MI or they did not make it clear they did not (albeit efforts to contact authors for clarification were made, with some success).

Upon subgroup analysis, our meta-analysis identified a statistical interaction between the incidences of in-hospital and 30-day death and the timing of angiographically-confirmed ST. In both analyses, patients experiencing early ST (within 30-days) appeared to have higher incidences of death than those experiencing very late ST (>1 year). It has been posited that these differences in fatality rates could be explained, at least partially, by ST timing-dependent differences in patient characteristics or variation in the pathophysiologic mechanisms of early and later occurring ST [7,18,32]. Our finding of no difference in early mortality among patients experiencing a thrombosed bare-metal or DES is consistent with the findings of prior studies [9,20,25]. Of note, we did not try to run subgroup analyses on the in-hospital and 30-day MI outcomes due to the small number of studies identified for these endpoints (N=5 and 3). Hopefully future larger studies or meta-analyses can address whether interactions exists between ST timing or stent type and post-ST MI outcomes.

The Agency for Healthcare Research and Quality’s (AHRQ’s) Healthcare Utilization Project (HCUP) estimated approximately 640,000 Americans were hospitalized to undergo coronary stent implantation in 2009 [1]. Therefore, despite the relatively low incidence of ST, the absolute number of patients experiencing ST is still quite large and likely to continue to grow as the use of coronary stents increase. If the median cost of treating a ST calculated by Reynolds and colleagues is inflated to a 2012US$ value ($17,686), and we assume a conservative ST rate of 1% [2], estimates of direct hospital costs alone of treating ST likely exceed $113 million per year. It is important to note, the study by Reynolds and colleagues [4] was a small study (N=23 subacute STs) including only patients receiving BMSs. Moreover, it did not estimate costs of ST due to delayed complications, outpatient healthcare utilization, and lost work productivity. Thus larger economic studies, which better match current stent use patterns and evaluate downstream costs of ST, are needed.

There are some limitations to our meta-analysis that merit further discussion. First, despite our best efforts to reduce heterogeneity between pooled studies through inclusion/exclusion criteria, high degrees of statistical heterogeneity in our base-case analyses were still present (I^2^>55% for all). Subgroup analysis suggested that some of this heterogeneity was explained by pooling studies including different ST-timing, but not the pooling of studies including different stent types. Due to the relative paucity of identified studies for most endpoints, we were not able to investigate other sources of heterogeneity on our results. A second limitation of our meta-analysis stems from the fact that the evaluation of post-ST outcomes was not the primary objective of many of the included studies. This likely explained some of the studies’ validity deficiencies noted, such as the failure to independently adjudicate ST and major adverse cardiovascular event outcomes. Next, since the majority of included studies in this meta-analyses evaluated ACS patients; our pooled incidence rates are likely most generalizable to this population. Finally, due to inconsistent or incomplete reporting of the time from angiographically-confirmed ST to the occurrence of death and/or MI, a number of studies had to be excluded from our meta-analysis. This latter limitation may in part explain the higher likelihood of publication bias noted some of our analyses. 

## Conclusions

Regardless of stent type used, the short-term consequences of coronary stent thrombosis (ST) appear significant. While stent type does not seem to affect the incidence of post-ST outcomes, an earlier occurrence of ST may be associated with higher mortality. 

## Supporting Information

File S1 **Supporting Information File.** Appendix S1, Medline Search Strategy. Appendix S2, Validity Assessment Tool Utilized for “Early Clinical and Economic Consequences of Coronary Stent Thrombosis.” Appendix S3, Academic Research Consortium Definitions and Timing Classifications of Coronary Stent Thrombosis.
(DOCX)
