# Supplementary material for: Short-Term Consequences of Angiographically-Confirmed Coronary Stent Thrombosis
Source: PLoS One. 2013 Oct 15;8(10):e77330. doi: 10.1371/journal.pone.0077330 (PMC3797034; doi:10.1371/journal.pone.0077330)
Supplement: File S1 — Supporting Information File. Appendix S1, Medline Search Strategy. Appendix S2, Validity Assessment Tool Utilized for “Early Clinical and Economic Consequences of Coronary Stent Thrombosis.” Appendix S3, Academic Research Consortium Definitions and Timing Classifications of Coronary Stent Thrombosis. (DOCX) [file pone.0077330.s001.docx]

**Appendix S1. Medline Search Strategy**

#1 (stent adj thrombosis).mp.

#2 (stent adj restenosis).mp.

#3 (stent adj occlusion).mp.

#4 (stent adj re-occlusion).mp.

#5 (stent adj reocclusion).mp.

#6 1 or 2 or 3 or 4 or 5

#7 limit 6 to humans

#8 limit 7 to yr="2000 -Current"

**#9 limit 8 to English language**

**Appendix S2. Validity Assessment Tool Utilized for “Early Clinical and Economic Consequences of Coronary Stent Thrombosis”***

1. **Were our meta-analysis’ *a priori* defined ST outcomes a primary objective of the study?**

Scored “yes” if incidence/rate of either MI or death and/or costs following ST was the primary outcome measure as stated in the study introduction/methods; “no” scoring is self-explanatory; “indeterminate” is not a choice.

1. **Were inclusion/exclusion criteria for the study clearly described?**

Scored “yes” if inclusion/exclusion criteria of the study were well described (need not be in any specific-part of the paper). Minimum criteria to be described include: 1) type of stent (DES or BMS) allowed, 2) reason for initial stent implant, 3) location/country of patient identification, 4) sampling time frame, and 5) requirement of DAPT. Scored “no” if **ALL** the above criteria are not addressed. “Indeterminate” is not a choice.

1. **Were consecutive and unselected cases of ST evaluated?**

Scored “yes” if evaluated ST cases were chosen on a consecutive (must be explicitly stated in the paper) **AND** unselected basis (NOTE: definition of ST should not be considered in the determination of “unselected” population; i.e., inclusion of only angiographically identified ST and exclusion of probable/possible cases should not result in scoring this criterion as “no”); scored “no” if the population was either non-consecutive or if the population was selected for the presence or absence of specific characteristics; scored “indeterminate” if patients were not selected for the presence or absence of specific characteristics, **BUT** the consecutive or non-consecutive nature of ST cases was not explicitly stated.

1. **Were data collected prospectively?**

Scored “yes” if data collection or study design was prospective (must be explicitly stated in the text to be score “yes’); scored “no” if data collection or study design was retrospective; scored “indeterminate” if prospective vs. retrospective timing of data collection cannot be reliably determined.

1. **Were the methods of data collection stated/described?**

Scored “yes” if methods for collecting ST and major adverse cardiovascular events (MACE) data were described clearly in the text. This should include a description of any tools and processes used; scored “no” if methods for data collection were not adequately described; “indeterminate” is not a choice.

1. **Was completeness of follow-up for ST outcomes described and adequate, and reasons for loss-of-follow up provided?**

Scored “yes” if **ALL** of the following criteria were met: 1) the paper accounted for the disposition (outcomes) of all identified ST cases **AND** 2) the disposition of at least 90% of identified ST cases was known and reported; “no” score is self-explanatory; “indeterminate” is not a choice.

1. **Were standard/valid/accepted definitions of ST and MI used?**

Scored “yes” if ST was defined using the ARC “definite” ST criteria (or equivalent) **AND** (if applicable) MI was defined at minimum as patients experiencing ≥2 of the following common diagnostic criteria:1) ischemic symptoms, 2) cardiac biomarker elevations and/or 3) EKG changes; scored “no” if either the ST definition or the MI definition (when applicable) were not as detailed above; scored “indeterminate” if definitions for ST and MI were not mentioned or described in enough detail to ascertain the exact criteria used to define these events.

1. **Were ST and MACE outcomes/events adjudicated?**

Scored “yes” if events (both ST and MACE) were adjudicated by an investigator or a core laboratory; scored “no” if the paper states events were not adjudicated (e.g., data came directly from chart review or reporting without verification); scored “indeterminate” if adjudication of outcomes was not mentioned.

1. **Were discussion/conclusions related to outcomes of ST consistent with respect to results?**

Scored “yes” if discussion/conclusions related to ST outcomes were consistent with respect to results; scored “no” if discussion/conclusions related to ST outcomes were inconsistent with results OR not discussed when ST outcomes was a primary objective of the research; scored “not applicable” if ST outcomes were not discussed AND were not the primary objective of the paper (note: not all papers evaluated ST outcomes as a primary endpoint as therefore were not expected to discuss this data in the discussion/conclusion sections)

1. **Were results related to ST outcomes compared/contrasted to other relevant studies?**

Scored “yes” if results related to ST outcomes were compared and/or contrasted to at least one previously conducted study on the same topic or state to their knowledge that no previous analyses existed; scored “no” if ST outcomes were not compared and/or contrasted to at least one previously conducted study on the same topic OR not discussed when ST outcomes was a primary objective of the research; scored “not applicable” if prior studies were not discussed AND ST outcomes were not the primary objective of the paper (note: not all papers evaluated ST outcomes as a primary endpoint as therefore were not expected to discuss this data in the discussion/conclusion sections).

1. **Were limitations (impact of biases) discussed?**

Scored “yes” if limitations (such as, but not limited to missing data, lack of adjudication, selection bias, non-consecutive or selected patients, and non-standardized definitions) were mentioned in the discussion/conclusions **AND** if impact of these biases on study results was discussed; “no” score is self-explanatory; “indeterminate” is not a choice.

1. **Was funding described? If funded by an extramural industry sponsor, was the role of the sponsor described?**

Scored “yes” if specific mention of funds, grant, or money for research and manuscript writing was described in the text **AND** if an industry sponsor was involved, the role/level of involvement was also stated; scored “no” if there is no mention of funding for the study (unfunded studies **MUST** state as much) or in case of industry funding, if the role of sponsor is not adequately.

***Note:** Studies are being assessed for their validity in answering this systematic review and meta-analysis’ specific hypotheses.

**Appendix S3. Academic Research Consortium Definitions and Timing Classifications of Coronary Stent Thrombosis**

**Definite Stent Thrombosis**

- - Angiographic or pathologic confirmation of partial or total thrombotic occlusion within the peri-stent region

AND at least ONE of the following, additional criteria:

- - Acute ischemic symptoms
  - Ischemic ECG changes
  - Elevated cardiac biomarkers

**Probable Stent Thrombosis**

- - Any unexplained death within 30 days of stent implantation
  - Any myocardial infarction, which is related to documented acute ischemia in the territory of the implanted stent without angiographic confirmation of stent thrombosis and in the absence of any other obvious cause

**Possible Stent Thrombosis**

- - Any unexplained death beyond 30 days

**Early Stent Thrombosis**

- - Any occuring from impant to 1 month

**Late Stent Thrombosis**

- - Any occuring > 1 month to 1 year after implant

**Very Late Stent Thrombosis**

- - Any occuring > 1 year after implant
